# Supplementary material for: The interactome of intact mitochondria by cross-linking mass spectrometry provides evidence for coexisting respiratory supercomplexes
Source: Mol Cell Proteomics. 2017 Dec 8;17(2):216–32. doi: 10.1074/mcp.RA117.000470 (PMC5795388; doi:10.1074/mcp.RA117.000470)
Supplement: Supplemental Data [file supp_RA117.000470_133922_0_supp_23978_fzffwf.zip › spectra_annotation/mito_spectra_annotation/423-1-4-1-13-1.pdf]

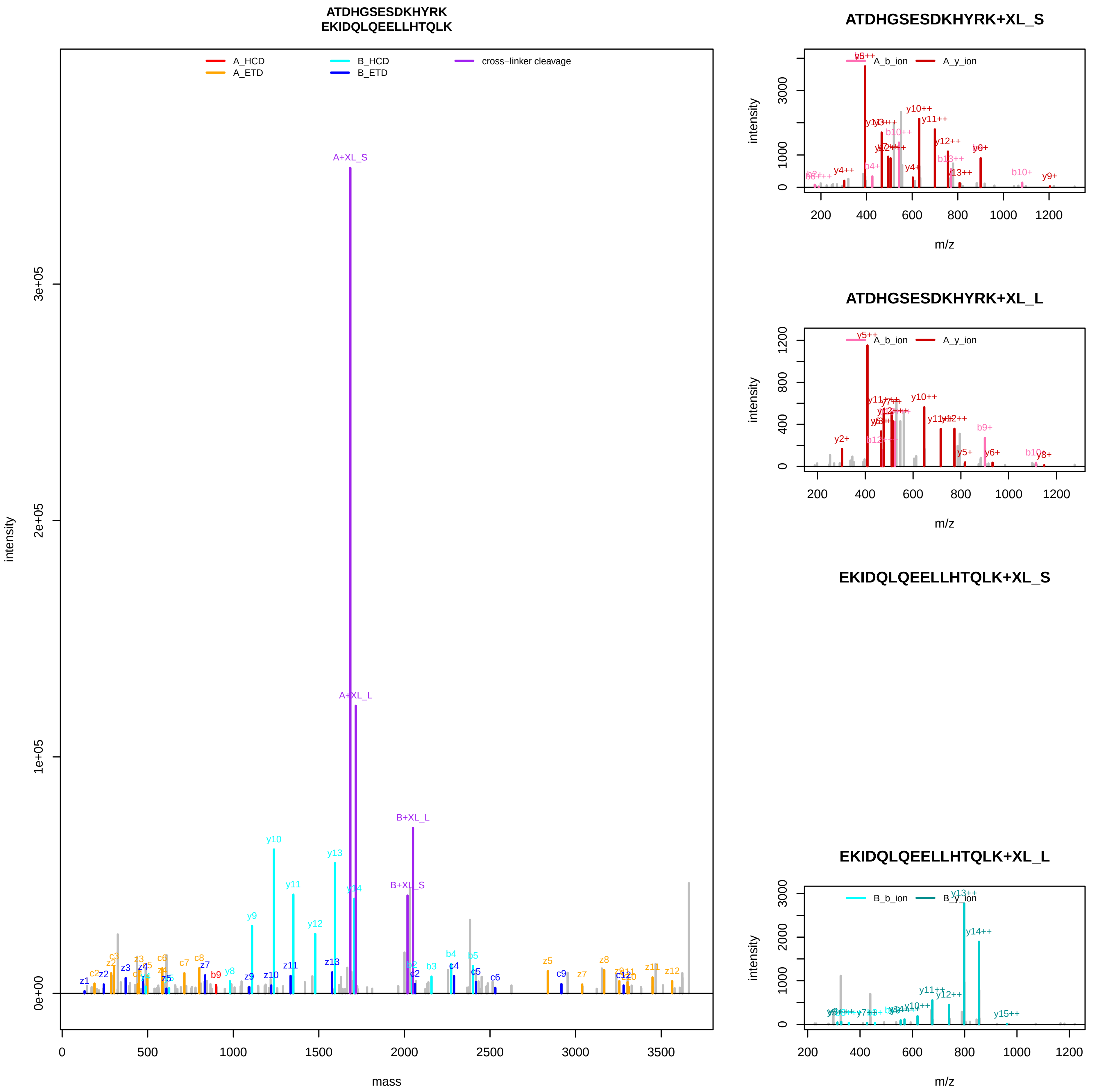

ATDHGSESDKHYRK
EKIDQLQEELLHTQLK
A_HCD
A_ETD
B_HCD
B_ETD
cross−linker cleavage
A+XL_S
A+XL_L
B+XL_L
B+XL_S
y10
y11
y12
y13
y9
y8
y14
z1
z2
z3
z4
z5
z7
z9
z10
z11
z13
c2
c3
c6
c7
c8
c9
c12
b9
b3
b4
b5
c4
c5
c6
z5
z7
z8
z11
z12
intensity
0e+00
1e+05
2e+05
3e+05
mass
0
500
1000
1500
2000
2500
3000
3500
ATDHGSESDKHYRK+XL_S
A_b_ion
A_y_ion
y5++
y10++
y11++
y12++
y4++
y4+
y6+
b10++
b13++
y13++
b10+
y9+
intensity
0
1000
3000
200
400
600
800
1000
1200
m/z
ATDHGSESDKHYRK+XL_L
A_b_ion
A_y_ion
y5++
y10++
y2+
y5+
y6+
b9+
b10+
y8+
intensity
0
400
800
1200
200
400
600
800
1000
1200
m/z
EKIDQLQEELLHTQLK+XL_S
EKIDQLQEELLHTQLK+XL_L
B_b_ion
B_y_ion
y13++
y14++
y11++
y12++
y10++
y15++
intensity
0
1000
2000
3000
200
400
600
800
1000
1200
m/z
